# Access to Specialized Medical Training in Spain and Determinants of Failure in the National Entrance Examination: Econometric Modeling Study

**DOI:** 10.2196/72440

**Published:** 2025-12-09

**Authors:** Montserrat Diaz-Fernandez, Mar Llorente-Marron, Victor Asensi

**Affiliations:** 1 Department of Quantitative Economics, University of Oviedo Oviedo Spain; 2 Department of Medicine, University of Oviedo Oviedo Spain

**Keywords:** medical profession, specialized medical training, access, gender, nationality, academic scale, COVID-19 pandemic

## Abstract

**Background:**

The process of accessing specialized medical training in Spain is a complex issue, involving not only the evaluation of medical knowledge acquired throughout university training but also the interaction of factors of a contextual and structural nature, which can influence the results obtained in the entrance examination. In this context, research on the variables that determine performance in this test is of great relevance form not only an academic but also a social and economic point of view. The interaction among factors such as academic performance, gender, nationality, and timing offers a unique opportunity to evaluate the functioning of the educational system at a critical moment in its recent history. Research that has focused specifically on access to specialized medical training has shown mixed results on how these factors impact examination performance.

**Objective:**

This study aimed to approximate the factors that determine failure in the entrance test for specialized medical training in Spain with the aim of better understanding the extent to which differences based on sex, nationality, and the context of the COVID-19 pandemic contribute to explaining such failure.

**Methods:**

We carried out econometric modeling of the final results obtained in the entrance examination to specialized medical training and identified the explanatory factors that determine the results, their relevance, effect, and significance. Econometric modeling provides a rigorous framework for estimating the causal effect of different variables on the final examination score. It helps identify not only which variables have an impact on performance but also to what extent they do so and under what conditions.

**Results:**

Based on the results obtained in the 2019-2021 test calls (7217 eliminated candidates), academic records (*P*<.001) and examination scores (*P*<.001), together with demographic factors including sex (*P*=.54) and nationality (*P*<.001), and calendar year (*P*<.001) were determinants of the behavior observed in the final results. Our results do not indicate whether being male or female favors or decreases the final grade obtained; however, being Spanish constitutes a relevant explanatory factor in our final results. The calendar effect, directly related to the COVID-19 pandemic, allows us to quantify the negative impact on the final results.

**Conclusions:**

This study investigated the impact of factors such as sex, nationality, and the COVID-19 pandemic on access to specialized medical training in Spain. Empirically, not being Spanish acts as an unfavorable fixed characteristic in the baseline econometric model, but it becomes favorable when considering the candidate's academic record. The impact of language is not perceived as a limiting factor; the test exclusively evaluates knowledge of medical content. The negative effects of the COVID-19 pandemic are visualized in the final scores.

## Introduction

The world’s population is growing, and life expectancy is increasing. This implies a greater consumption of health services, which is essential for guaranteeing social well-being. However, recourses are limited. In this context, adequate planning for training of health professionals to meet the current and future needs of the population is unavoidable. In light of this, health care resource planning studies have been conducted [1,2].

The Spanish health care system has serious problems due to high levels of life expectancy and medical insufficiency. As a result, the increase in life expectancy, population growth, and the number of people in confinement together contribute to the increase in the demand for health services [3]. Spain has experienced a rapid decline in birth rate and population growth, which has caused a significant demographic change that has affected the country’s present and future. In 2022, life expectancy in Spain was 3 years longer than the average within the European Union (EU). Currently, 20% of the population of Spain is older than 65 years.

Health spending in Spain has grown in recent years, although it is lower than the EU average. In 2019, Spain dedicated 9.1% of its gross domestic product to health spending, that is, 0.8 percentage points less than the EU average, with a per-capita spending of €2488 (US $ 2885.15), which is 30% lower than the EU average (€3523 [US $ 4085.46]) [4]. The number of doctors per inhabitant in Spain (4.33 doctors per 1000 inhabitants) exceeds the average (3.6 doctors per 1000 inhabitants) in OECD (Organisation for Economic Co-operation and Development) countries. However, doctor shortages and supply rigidities remain key problems. In this debate, the difficulties in reconciling family life in a female-dominant sector play a relevant role, as does the impact of the mass retirement of many professionals and the expatriation of talent to other countries.

The medical profession has experienced significant social change in recent decades, with a greater presence of women at all levels and specialties. This feminization process has occurred in most high-income countries and is expected to continue in the future [5-9]. In Spain, while 2 out of every 3 doctors who retire are men, 2 out of every 3 physicians who join are women. Currently, 7 out of 10 students enrolled in medical schools are women, and a similar metric is observed for resident physicians (6 out of every 10 residents are women) [10]. However, the aging of the health care workforce and the flight of talent to other countries can threaten the quality and viability of Spain’s health care system [3]. The average age of active physicians in 2021 was 49.76 years, with a bimodal distribution of those in the age groups of 45-64 years (37.75%) and 35-54 years (38.78%). The perception of professional opportunities influences the mobility of physicians, who may feel demotivated by their current situation or attracted by other options [11]. The outflow of physicians from Spain to other countries in search of better working conditions, salaries, and professional recognition has continued in recent years, despite the COVID-19 pandemic. According to data from the Collegiate Medical Organization, 2504 certificates of suitability for work abroad were issued in 2021, which was similar to the number issued in 2020. Most of the chosen destinations are EU countries where better options are offered, especially with regard to working conditions and compensation. On the other hand, the arrival of foreign doctors in Spain is related to the economic cycle of public health spending, which has suffered cuts during the economic crisis [11,12].

Public health constitutes a fundamental aspect of the development and quality of life of any country. Different indicators can be used for evaluation (patients’ rights, waiting lists, prevention programs, free access, etc). The universal right to health in Spain is guaranteed through the National Health System (NHS), which covers 96.5% of health care services. The NHS offers free, quality services to all citizens throughout their lives and contributes to the state of well-being. Spain stands out as one of the countries with the best health systems worldwide.

Technological capacity and highly qualified human capital constitute strengths of the NHS. The implementation and official recognition of the medical specialization system in Spain through the Medical Intern Residency (MIR) program as a means of accessing the specialist title has led to decisive advances in Specialized Health Training (SHT) tests, which serve as a mark of prestige and recognition for NHS professionals [13]. The SHT based on the residency system consists of a programmed, supervised, and paid clinical practice that pursues the progressive assumption of responsibilities throughout the training period. The SHT aims to train health professionals in the knowledge, techniques, and skills of each specialty, guaranteeing comprehensive and efficient care for users. The assignment of candidates to MIR placements by specialty and hospital center is based on an ordered election based on the results of a national examination with multiple-choice questions that are determined based on the principles of merit and ability and the academic scale of the degree in medicine [14,15]. The choice of specialty is therefore based on the principle of vertical equity by allowing the most productive candidates to have priority in the choice of the SHT program using the ranking position as a proxy variable. Certification examinations play a fundamental role in terms of social responsibility, as they provide objective and transparent criteria for assessing candidates' performance [16-18].

Various studies have shown that factors such as sex, nationality, or the COVID-19 pandemic have an impact on the final results of participants who pass the SHT access test in Spain [7,19]. However, not everyone passes the test; approximately 1 in 5 participants are eliminated from the process during the access attempt. However, the literature does not include the profile of eliminated applicants who do not exceed the access threshold. The shortage of human and material resources in some areas of the country, labeled medical deserts, makes access to quality health care appropriate to the needs of the population difficult. Health planning is a complex process that requires evaluating health needs, establishing objectives and strategies to improve health, and allocating health resources efficiently and equitably. Its purpose is to offer solutions to health problems that affect people through actions to prevent, treat, control, or eliminate diseases. To do this, not only conventional health services but also the social, economic, and material aspects that influence health must be considered [20]. Studying whether there are significant differences among the factors that determine success or failure in access to the SHT can be useful in the context of a worrying shortage of health professionals.

In this study, we aim to examine the factors that determine the performance of candidates in terms of access to the SHT, which is a demanding test that provides access to specialized training in health care in Spain. However, not everyone manages to overcome it, leaving some outside the selection process. What factors influence success or failure? Are there differences based on sex or nationality? Has the COVID-19 pandemic had an influence on the outcomes? Based on the data of applicants who did not achieve the minimum grade between the 2019 and 2021 test calls, we explore potential differences based on sex, nationality, and the context of the COVID-19 pandemic. We intend to determine the explanatory factors that are associated with the final score obtained and offer relevant information for health planning, which must respond to the needs and expectations of citizens in terms of health, as well as the social, economic, and material progress of people. This work can help optimize access to the SHT and mitigate medical desert areas in Spain.

Does the use of traditional scoring metrics for resident recruitment create racial and gender bias [21]? Our objective is to identify the explanatory factors that approximate the final score on the national entrance examination to access the SHT. We intend to analyze quantitatively to what extent the examination, sociodemographic factors, and calendar data explain the results of the MIR examination. To do so, we will use an econometric approach that will allow us to establish a causal relationship between the results obtained and the variables that determine them.

## Methods

### Data

The statistical information used in this study was obtained from the final list of results of the selective medical examinations, prepared by the Ministry of Health for the annual calls during the 2019-2021 period [22]. The recruitment process was conducted through a national public call, in which applicants voluntarily register and meet basic administrative and academic requirements. The registration forms collect demographic information of the applicants, examination data, and details of the test schedule, allowing systematic tracking of each stage of the process. The analyzed period corresponds to the 3 examinations conducted between 2020 and 2022, with complete information available for all variables across all observations and no missing data. For the analysis, only the number of candidates who did not pass the examination was considered, defined as those scoring below the minimum established threshold (35% of the arithmetic mean of the 10 highest exam scores). In the 2019, 2020, and 2021 calls, the cutoff scores were 157, 156, and 177 points, respectively. In the 2022 call, the cutoff criterion was adjusted to 25% of the arithmetic mean of the 10 highest examination scores, lowering the minimum passing score to 113 points. In total, 7217 observations were analyzed: 2796 from 2019, 2526 from 2020, and 1895 from 2021.

### Ethical Considerations

This study did not require review or approval by an institutional ethics committee, as it relies exclusively on publicly available, anonymized, and aggregated secondary data.

In accordance with international research ethics guidelines (such as the Declaration of Helsinki), the recommendations of the Spanish Ministry of Science and Innovation’s Research Ethics Committee for studies using public data, and JMIR’s editorial policies, studies using such data are considered exempt from formal ethical review.

### Procedure

The final grade obtained, that is, the score, is obtained as a weighted average of the test examination (90%) and academic records (10%). This variable approximates the final result in which factors specifically related to the examination—valid answers, validity, academic records, scale, demographic factors, sex, nationality and language, and the calendar factors MIR_2020 and MIR_2021—contribute to the observed behavior of the final result (Table 1). The effect of the calendar year is approximated from the annual data corresponding to the calls made between 2019 and 2021 and the variables MIR_2019, MIR_2020 (MIR_2020=1), and MIR_2021 (MIR_2021=1), with the 2019 call serving as the control category.

**Table 1:** Selected variables used in the study.

Indicators, variables, and their definitions		Expected sign
**Test**		The expected sign of this variable is positive
	Valid: quantitative variable indicating the total number of correct answers obtained by each candidate	
	Scale: quantitative variable obtained by multiplying by 10 the evaluation of each candidate's file and dividing the product by the average of the 10 best examinations of the corresponding exercise	
**Demographic factors**		A priori; the expected sign of this variable can be + or –
	Sex: dichotomous variable that takes the value 0 if the applicant is female, 1 otherwise	
	Nationality: dichotomous variable that takes the value 0 if the applicant is of Spanish nationality, 1 otherwise	
	Language: dichotomous variable that takes the value 0 if the applicant communicates in Spanish, 1 otherwise	
**Calendar**		A priori; the expected sign of this variable can be + or –
	MIR_2020^a^: dichotomous variable that takes the value 1 if the sample observation corresponds to the 2020 call, otherwise 0	
	MIR_2021: dichotomous variable that takes the value 1 if the sample observation corresponds to the call for the year 2021, 0 otherwise	

^a^MIR: Medical Intern Residency.

The specification of the model to be used requires the determination of the variables and functional relationship to be considered:

*Score_i_* = *f* (*Valid_i_*, *Scale_i_*, *Sex_i_*, *Nationality_i_*, *Language_i_*, *MIR*2020*_i_*, *MIR*2021_i_, *u_i_*)

where, for the *ith* observation, the variable that includes the final grade obtained in the test, *Score*, denotes the dependent variable, and *u* the random disturbance term. Linear regression models were used and estimated using the ordinary least squares (OLS) method to explain *Score* based on a set of explanatory variables. All explanatory variables were selected based on theoretical relevance and data availability. Additionally, interaction terms were included where supported by prior literature or exploratory analysis. Multicollinearity was assessed using the linear correlation matrix of the explanatory variables, which showed no signs of high multicollinearity. However, when heteroskedasticity is present—meaning that the variance of the error terms is not constant—the SEs produced by OLS may be unreliable, compromising the validity of statistical inference. To address this, we use heteroskedasticity-robust SEs, specifically the Huber-White-Hinkley estimator, which adjusts the variance-covariance matrix to yield consistent SE estimates even under nonconstant error variance. This ensures more accurate hypothesis testing. Moreover, the use of this multivariate approach enables the estimation of each predictor’s unique contribution while accounting for the influence of other variables, which is crucial for reducing potential biases due to confounding—particularly in nonexperimental research settings.

To make the sprint and background effects visible, taking into account the gender of the student as well as the impact that the COVID-19 pandemic had on the results for each sex, interaction terms of the sex variable with the valid, scale, MIR_2020, and MIR_2021 variables are included in the model. Similarly, to make the effect of the nationality variable visible, interaction terms are included for the valid, scale, MIR_2020, and MIR_2021 variables.

## Results

Table 2 shows the descriptive statistics of the variables considered based on a sample of 7217 observations. For qualitative variables, information is made visible on the number of observations that satisfy the analyzed characteristics and their relative frequency. For the quantitative variables, the mean, median, IQR, minimum value, maximum value, and sample SD are used. Statistical analysis and model estimation were performed using the EViews program (IHS Global Inc) and our predetermined α level was .05.

**Table 2:** Variable definition and summary statistics (2019-2021; N=7217). This is our own elaboration based on Ministry of Health 2022 data.

Indicators				Values
**Demographic factors**				
	**Qualitative variables, n (%)**			
		**Sex**		
			Male	4155 (57.57)
			Female	3062 (42.43)
		**Nationality**		
			Spanish	3409 (47.24)
			Non-Spanish	3808 (52.76)
		**Language**		
			Spanish	6468 (89.62)
			Not Spanish	749 (10.37)
**Calendar**				
	**MIR^a^ call, n (%)**			
		MIR_2019		2796 (38.74)
		MIR_2020		2526 (35.00)
		MIR_2021		1895 (26.26)
**Test**				
	**Quantitative variables**			
		**Valid**		
			Mean (SD)	68.05 (12.44)
			Median (IQR)	69 (60-77)
			Range	0-94
		**Scale**		
			Mean (SD)	6.11 (1.13)
			Median (IQR)	6.2 (5.00-7.04)
			Range	4.37-5.00

^a^MIR: Medical Intern Residency.

During the selected period, the total number of eliminated candidates decreased. Of a total of 7217 eliminated candidates who attempted to earn an SHT place, 38.74% (n=2796) corresponded to the 2019 call. In the following calls, the weight was slightly lower. Between the initial and final calls, the total number of eliminated candidates decreased by 10.48 percentage points. Of the total sample, 57.57% (n=4155) of the eliminated candidates were women and 47.24% (n=3409) were Spanish. The native language of 1 in 10 of the eliminated applicants was not Spanish. The median number of valid responses and scale scores during the examined period was 69 (IQR 60-77) correct answers and 6.2 (IQR 5.00-7.04) points, respectively. The linear correlation matrix of the explanatory variables shows no signs of high multicollinearity, indicating that the independent variables are not excessively correlated with each other and can be reliably included in the regression model (Table 3). It also indicates that during the period analyzed, the direction of the linear association between the qualitative factors sex and nationality and each of the quantitative factors—validity and scale—was inversely proportional (*r*_Sex-Valid_=–0.0202; *r*_Sex-Scale_=–0.0505; *r*_Nationality-Valid_=–0.0766; *r*_Nationality-Scale_=–0.0109).

**Table 3:** Correlation coefficients determined using observations 1 to 7217. The critical value at 5% (2-tailed) was 0.0231 for N=7217.

	Nationality	Valid	Scale	Sex	MIR_2020	MIR_2021	Sex × valid	Sex × MIR_2020	Sex × MIR_2021	Nationality × valid	Nationality × scale	Language
Nationality	1	–0.0766	–0.0109	0.0032	–0.0492	0.0136	–0.0066	–0.0105	–0.0103	0.9652	0.9613	0.2912
Valid	—^a^	1	0.2013	–0.0202	–0.2033	0.3388	0.1397	–0.1369	0.2028	0.1171	–0.0359	–0.0444
Scale	—	—	1	–0.0505	0.0601	0.1388	–0.0146	0.0095	0.0688	0.0271	0.2005	–0.041
Sex	—	—	—	1	–0.001	0.0032	0.9709	0.486	0.4139	0.0013	–0.0069	0.0186
MIR_2020	—	—	—	—	1	–0.4379	–0.0352	0.5686	–0.2607	–0.089	–0.0328	–0.0192
MIR_2021	—	—	—	—	—	1	0.0577	–0.249	0.5955	0.0772	0.0461	–0.0079
Sex × valid	—	—	—	—	—	—	1	0.426	0.478	0.0214	–0.0102	0.0072
Sex × MIR_2020	—	—	—	—	—	—	—	1	–0.1482	–0.0363	–0.0082	–0.0026
Sex ×MIR_2021	—	—	—	—	—	—	—	—	1	0.0271	0.0066	–0.0086
Nationality × valid	—	—	—	—	—	—	—	—	—	1	0.9414	0.2729
Nationality × scale	—	—	—	—	—	—	—	—	—	—	1	0.2672
Language	—	—	—	—	—	—	—	—	—	—	—	1

^a^Not applicable.

Following the approach adopted for the analysis of applicants regarding access to the SHT who passed the MIR examination [19], Table 4 shows the results corresponding to the eliminated applicants. The model is estimated with SEs and robust covariances given the cross-sectional nature of the support. The regression coefficient is globally significant (*F*_9,7201_=4038.137), and the collinearity indicators do not show severe linear association problems. These results indicate that not all the selected factors constitute relevant determinants that help to explain the observed behavior of the final result obtained.

**Table 4:** Regression model for the final score (2019-2021; MIR_2019: n=2796; MIR_2020: n=2526; MIR_2021: n=1895; MIR: Medical Intern Residency). This is our own elaboration based on the Ministry of Health 2022 data.

Dependent variable: score	β coefficient	*P* value
Constant	28.7606	<.001
Valid	0.6012	<.001
Scale	0.9972	<.001
Sex	0.3186	.54
Nationality	–0.6178	<.001
MIR_2020	–0.3359	<.001
MIR_2021	–6.2058	<.001
Sex × valid	–0.0049	.48
Sex × MIR_2020	–0.0574	.65
Sex × MIR_2021	0.0699	.65
Adjusted *R*	0.8344	—^a^
*F* test (*df*)	4038.137 (9,7201)	<.001

^a^Not applicable.

The effects on the final results of the examination component and academic records are determined through the validity and scale variables. The effect on the final test result in both cases is shown to be directly proportional and significant. The effect of the applicant's sex is approximated by the sex variable and its interaction with the variable that approximates the number of correct answers provided on the examination. In both cases, the effect on the final result is not perceived as significant. The nationality of the applicant is approximated by the nationality variable, and its effect is found to be significant. The results obtained show the differential effect on the final result between Spanish (control category) and non-Spanish individuals. The difference in the total score obtained between a Spanish and non-Spanish eliminated candidate, ceteris paribus, is 0.6178 lower for the latter. The contribution of the calendar year is captured by the variables MIR_2020 and MIR_2021, which collect the observations corresponding to the tests carried out in March 2021 and January 2022, respectively. The result obtained is negative and significant in both instances. The final results of the tests carried out in 2021 and 2022, ceteris paribus, are 0.3116 and 6.2355 points lower than those of the control category, respectively. The effects of the interaction of the calendar factor and the sex factor are collected through the interaction variables sex × MIR_2020 and sex × MIR_2021, whose effect on the final result of the call is not perceived as being significant.

During the period analyzed, 1 in 4 applicants were not Spanish and neither were 45.24% (n=3265) of the total number of eliminated applicants, of which 81% came from South America. Table 5 explores the impact of the origin of the eliminated candidates on the final result. The effect of origin and its interaction with both the examination and academic records are analyzed through the variables nationality, nationality × valid, and nationality × scale, respectively. Three alternative models are estimated to approximate the behavior of the final test result. Model 1 incorporates the variable nationality × scale, model 2 adds the interaction with the examination through the nationality × valid variable, and model 3 adds the nationality × valid variable. Based on the results obtained, the model chosen is the one that offers the greatest predictive capacity and incorporates the smallest number of regressors. Based on the goodness-of-fit test, model 2, which simultaneously incorporates the interaction of nationality with the variables validity and scale, registered the highest value (0.8390). However, model 1 minimizes the values of the Akaike information criterion (27721.32), the Schwart criterion (27797.04) and the Hannan-Quinn criterion (27747.37); it also incorporates a smaller number of explanatory factors. The regression coefficient is globally significant (F*_10,7206_=3660.368), and the collinearity indicators do not show severe linear association problems.

**Table 5:** Regression model for the final score (2019-2021; MIR_2019: n=2796; MIR_2020: n=2526; MIR_2021: n=1895; MIR: Medical Intern Residency). This is our own elaboration based on the Ministry of Health 2022 data.

Dependent variable: score	Model 1^a^			Model 2^b^			Model 3^c^	
	β coefficient	*P* value	β coefficient		*P* value	β coefficient		*P* value
Constant	28.7631	<.001	28.7988		<.001	28.7943		<.001
Valid	0.601825	<.001	0.5967		<.001	0.5972		<.001
Scale	0.9892	<.001	0.9952		<.001	0.9953		<.001
Sex	0.2701	.60	–0.0561		.92	0.0261		.95
Nationality	–1.4682	<.001	–6.8459		<.001	–6.7054		<.001
MIR_2020^d^	–0.3425	<.001	–0.3259		<.001	–0.3261		<.001
MIR_2021	–6.2203	<.001	–6.1005		<.001	–6.1010		<.001
Sex × valid	–0.0044	.53	7.0636×10^–^^05^		.99	–0.0006		.89
Sex × MIR_2020	–0.0470	.71	–0.0452		.72	–0.0517		.68
Sex × MIR_2021	0.0741	.63	0.0094		.95	0.0165		.91
Nationality × scale	0.1362	.003	0.0214		.64	—^e^		—
Nationality × valid	—	—	0.0791		<.001	0.0791		<.001

^a^Adjusted *R*=0.8342; *F*_10,7200_=3660.368; *P*<.001; Akaike information criterion=27721.32; Schwarz criterion=27797.04; Hannan-Quinn criterion=27747.37.

^b^Adjusted *R*=0.8390; *F*_11,7199_=3417.508; *P*<.001; Akaike information criterion=27769.02; Schwarz criterion=27851.62; Hannan-Quinn criterion=27797.44.

^c^Adjusted *R*=0.8388; *F*_10,7200_=3154.413; *P*<.001; Akaike information criterion=27773.24; Schwarz criterion=27848.96; Hannan-Quinn criterion=27799.30.

^d^MIR: Medical Intern Residency.

^d^Not applicable.

The impact on the final test score of the explanatory factors validity and scale is, in both cases, directly proportional and significant. The sex and sex × valid variables approximate the effect on the final result of the qualitative factor sex and its interaction with the examination component, respectively, none of which are perceived as significant. The effects that origin and academic records have on the final result are approximated through the variables nationality and nationality × scale, respectively. The impact of both variables is significant, although the signs of the results are opposite in nature. The difference in the total score obtained between Spanish and non-Spanish eliminated candidates is, ceteris paribus, 1.4682 points lower for the latter. However, the differential effect corresponding to the scale between the final result of a Spanish and non-Spanish eliminated candidate, ceteris paribus, is 0.1362 points greater for the non-Spanish candidate.

The impact that the calendar year has on the final result is collected by the variables MIR_2020 and MIR_2021, which group together the observations corresponding to the tests carried out in March 2021 and January 2022, respectively. The result obtained is negative and significant in both references. The final results of the tests carried out in 2021 and 2022 are, ceteris paribus, 0.3425 and 6.2203 points lower than those of the other years, respectively. The effects of the interaction of the calendar factor and the sex factor through the variables sex × MIR_2020 and sex × MIR_2021 did not significantly impact the final result.

Table 6 shows the estimation of the standardized coefficients, which facilitates the ranking of the relative weight of the explanatory variables in the model over the dependent variable. According to this criterion, the dominance of the examination variable in the data distribution is visualized in the final result through the variable that includes the number of correct answers provided on the test. Next, the weights of the variables that approximate the calendar year represent the second and sixth positions in the rankings, MIR_2021 and MIR_2020, respectively, and the academic records obtained through the scale variable rank third. The fourth and fifth positions are occupied by the origin of the applicant and its interaction with the academic record, respectively. The weight of the sex factor completes the distribution.

**Table 6:** Standardized β coefficients. This is our own elaboration based on the Ministry of Health 2022 data.

Variable	Standardized β coefficient	Order position
Constant	—^a^	—
Valid	0.9639	1
Scale	0.1451	3
Sex	0.0172	8
Nationality	–0.0944	4
MIR_2020^b^	–0.0210	6
MIR_2021	–0.3525	2
Sex × valid	–0.0194	7
Sex × MIR_2020	–0.0018	9
Sex × MIR_2020	0.0024	10
Nationality × scale	0.0544	5

^a^MIR: Medical Intern Residency.

Of a total of 7217 candidates eliminated, 3808 physicians of non-Spanish origin did not pass the SHT access test, with 2639 coming from South America. The native language of 749 of the eliminated test candidates was not Spanish. The consideration of language as a possible limiting factor for taking the test is only reflected in the examination score by determining the total number of correct answers. Table 7 shows the results of the examination score, namely, exam_score, based on the validity and language variables. The regression coefficient is globally significant, and the collinearity indicators do not show linear association problems. The effect of the valid variable on the examination score is shown to be directly proportional and significant. However, the effect of the explanatory factor of language is not perceived as significant.

**Table 7:** Examination score (2019-2021; MIR_2019: n=2796; MIR_2020: n=2526; MIR_2021: n=1895; MIR: Medical Intern Residency). This is our own elaboration based on the Ministry of Health 2022 data^a^.

Dependent variable: examination score	β coefficient	*P* value
Constant	20.9447	<.001
Valid	0.5294	<.001
Language	−0.0494	.75

^a^Adjusted *R*=0.6603; *F*_2,7214_=7013.959; *P*<.001.

## Discussion

This study fills an important gap in the literature by focusing on the profile and factors influencing candidates who fail to gain access to SHT in Spain, which is approximately 1 in 5 applicants. Using econometric modeling of data from the 2019-2021 MIR examination calls, we identified the key quantitative and qualitative variables affecting the final scores of those excluded, including examination results, academic records, nationality, gender, and the impact of the COVID-19 pandemic.

Some studies have analyzed the factors that determine the results of SHT entrance examinations in Spain [7,19]. However, there is little information on the profile of applicants who do not gain access and are ultimately excluded from the process, that is approximately 1 in 5 applicants. Knowing the differences between the factors that determine the success/failure of access to the SHT could favor the management of health care in the context of a scarcity of human resources.

Our empirical exercise focused on econometrically modeling the final MIR examination results for participants eliminated from the process during 2019-2021. The econometric approach allows causal analysis to be carried out by identifying and classifying the explanatory factors that help elucidate the final scores and their relevance, effect, and meaning [23]. Although the final results combine examination scores and academic records, they also reflect examinees’ interaction with real-world environments, justifying the econometric integration of both quantitative and qualitative factors such as sex, nationality, and examination calendar.

The study’s findings reveal significant differences between Spanish and non-Spanish applicants, with a persistent structural disadvantage in the performance of the latter. Among the relevant factors defining the final score, the validity and scale variables—corresponding to the so-called sprint effect and background effect, respectively—stand out [7,24]. An additional valid examination answer or one more point in the academic record, ceteris paribus, increases the final score, though the sprint effort (ie, examination performance) outweighs the background effort (ie, academic record) in standardized terms. This dominance aligns with that reported in studies emphasizing differential risk exposures by gender [25,26], though these variables alone do not clearly indicate success or failure, and their relationship remains coincidental [19].

Regarding sociodemographic factors, sex did not have a significant effect on final results, contrary to some previous studies suggesting male candidates historically achieve higher scores [15,27-29]. Furthermore, the interaction between sex and examination validity did not demonstrate a consistent effect. In contrast, nationality emerged as a significant explanatory factor: being non-Spanish carried a fixed, unfavorable impact on final scores, ranking fourth in standardized importance after academic record. The Ministry of Health requires degree homologation based on MIR examination success for professional practice in Spain [13]. Approximately 1 in 4 candidates during the study period was non-Spanish, with 45.24% (n=3265) of these candidates having been eliminated.

Importantly, the interaction between nationality and academic record was positive and significant, suggesting that strong academic backgrounds can partly compensate for structural disadvantages faced by non-Spanish applicants. This finding may point to systemic barriers such as degree recognition issues or implicit biases rather than linguistic obstacles, as native language was not a significant factor despite 749 eliminated non-Spanish candidates having a different native language [19,30,31].

Another key conclusion relates to the negative impact of the COVID-19 pandemic on final grades. The model’s calendar variables show that candidates from the 2020 and 2021 examination calls scored significantly lower than the prepandemic cohort. Pandemic-induced disruptions—including rapid shifts to virtual or hybrid learning and elevated stress—impaired examination preparation [32-36]. Notably, the interaction between sex and examination year was not significant.

From a causal perspective, examination results, calendar year, academic records, and sociodemographic factors are key determinants of the final scores of excluded candidates. While sex remains a determinant for successful candidates regardless of nationality [19], among those excluded, nationality plays a more prominent role. This highlights the importance of internationalization in the medical profession as a health policy factor to balance supply and demand in Spain’s health system. The regulated SHT program aims to ensure quality and comprehensive care, and immigration of doctors prepared for the MIR examination may help alleviate short-term workforce deficits and increase labor market flexibility.

In light of these conclusions, it is essential to analyze, evaluate, and prioritize the factors that influence final scores and the allocation of places. This approach can serve as a basis for designing policies that improve equity and efficiency in access to the health care system. The observed disparities underscore the need for targeted support mechanisms for internationally trained candidates. These may include structured mentoring programs, individualized guidance, and orientation strategies aimed at improving their integration and performance. Furthermore, in anticipation of future crises, educational authorities should consider implementing academic recovery measures and psychological support systems to ensure more balanced and inclusive preparation environments.

To address the underlying causes of these disparities, it is also advisable to implement specific support policies—such as guidance programs, tutoring or integration strategies—for candidates trained abroad. Key policy actions could include reviewing and streamlining the recognition process for foreign medical degrees, creating mentorship networks that connect international candidates with experienced professionals in the Spanish health care system, and developing crisis-response protocols that include flexible examination preparation resources and integrated emotional support services for all applicants.

By addressing these structural challenges, the Spanish health care system can take meaningful steps toward a more inclusive and resilient process for accessing specialized medical training.

One of the main limitations of this study lies in the nature of the data used. The dataset provided by the Ministry of Health is fully anonymized, which prevents any longitudinal tracking of individual candidates across different examination sessions or stages of the selection process. As a result, it is not possible to analyze repeated attempts by the same individuals or to evaluate changes in performance over time. This limits the ability to draw conclusions about the evolution of candidates' profiles or the cumulative impact of training and preparation efforts. Despite this constraint, the study offers a robust cross-sectional analysis of a large and representative sample of excluded candidates over 3 MIR calls, contributing valuable insights into a largely understudied population.

A promising future line of research involves conducting independent qualitative studies to complement the quantitative findings. Since the current dataset is anonymized and does not allow for the identification of individual applicants, future studies could use interviews, focus groups, or surveys with a representative sample of excluded candidates. This would provide deeper insights into their personal experiences, perceived barriers, and contextual challenges such as preparation conditions, psychological factors, or socioeconomic constraints, which may not be fully captured through econometric modeling alone.

## Data Availability

The datasets generated during this study are available from the corresponding author on reasonable request.

## Abbreviations

EU: European Union
MIR: Medical Intern Residency
NHS: National Health System
OECD: Organisation for Economic Co-operation and Development
OLS: ordinary least squares
SHT: Specialized Health Training

## References

[1] Roberfroid D, Leonard C, Stordeur S (2009). Physician supply forecast: better than peering in a crystal ball?. Hum Resour Health.

[2] Safarishahrbijari A (2018). Workforce forecasting models: A systematic review. Journal of Forecasting.

[3] Szabo S, Nove A, Matthews Z, Bajracharya A, Dhillon I, Singh DR, Saares A, Campbell J (2020). Health workforce demography: a framework to improve understanding of the health workforce and support achievement of the Sustainable Development Goals. Hum Resour Health.

[4] (2021). España: Perfil Sanitario del país 2021. Organisation for Economic Co-operation and Development.

[5] Kwon E (2016). “For Passion or for Future Family?” Exploring factors influencing career and family choices of female medical students and residents. Gend. Issues.

[6] Riska E (2011). Gender and medical careers. Maturitas.

[7] Rodriguez Santana I (2021). Becoming a resident in a high demanded medical specialty: an unequal race? Evidence from the Spanish resident market. Hum Resour Health.

[8] Babaria P, Abedin S, Berg D, Nunez-Smith M (2012). "I'm too used to it": a longitudinal qualitative study of third year female medical students' experiences of gendered encounters in medical education. Soc Sci Med.

[9] Heru AM (2005). Pink-collar medicine: Women and the future of medicine. Gend. Issues.

[10] Estadística de Estudiantes. Ministerio de Ciencia, Innovación y Universidades.

[11] (2019). Estimación de la oferta y demanda de médicos especialistas: España 2018-2030. Universidad de Las Palmas de Gran Canaria.

[12] Pastor-Bravo M, Nelson S (2019). Migration of Latin American nurses to Spain 2006-2016: a case study. Int Nurs Rev.

[13] Pardo JF (2021). Formación sanitaria especializada: un cambio es inevitable. Educación Médica.

[14] Aranda Briones B (2024). Advancing the delimitation of electronic voting: the saga continues with a new trilogy. Annotated Spanish Parliamentary Case Law.

[15] Bonillo A (2012). [Access tests to specialized health training for doctors and other healthcare professionals in Spain: examining the exam and the examined candidates]. Gac Sanit.

[16] Kelleher M, Schumacher DJ, Zhou C, Kwakye D, Santen SA, Warm E, Kinnear B (2025). Public board acore reporting undermines holistic review for residency selection. J Gen Intern Med.

[17] Muecke T, Rao A, Walker H, Tinnion J, Jesudason D, Bacchi S, Casson R, Chan WO (2024). Specialists of tomorrow: an umbrella review of evidence supporting criteria used in medical and surgical specialty training selection processes. Discov Educ.

[18] Muecke T, Bacchi S, Casson R, Chan WO (2024). Building the workforce of tomorrow: The weighting of rural exposure in standardised curriculum vitae scoring criteria for entrance into Australian specialty training programs. Aust J Rural Health.

[19] Díaz-Fernández Montserrat, Llorente-Marrón Mar, Cocina-Díaz Virginia, Asensi V (2023). COVID-19 and access to medical professional careers: Does gender matter?. Int J Environ Res Public Health.

[20] Lombardi CV, Chidiac NT, Record BC, Laukka JJ (2023). USMLE step 1 and step 2 CK as indicators of resident performance. BMC Med Educ.

[21] Lai SH, Suarez-Pierre A, Jaiswal K, Travis C, Steward L, Nehler M, Zweck-Bronner S, Christian N (2023). Implementation of a holistic review process of US allopathic medical students eliminates non-comparable metrics and bias in general surgery residency interview invitations. J Surg Educ.

[22] Formación Especializada. Ministerio de Sanidad.

[23] Nuñez LF (2011). Econometría de evaluación de impacto. Economía.

[24] Dávila-Quintana CD, López-Valcárcel BG, Barber P, Ortún V (2015). El baremo académico en el acceso a la formación médica especializada en España. FEM (Ed. impresa).

[25] Gneezy U, Niederle M, Rustichini A (2003). Performance in competitive environments: Gender differences. The Quarterly Journal of Economics.

[26] Niederle M, Vesterlund L (2010). Explaining the gender gap in math test scores: The role of competition. Journal of Economic Perspectives.

[27] Dayal A, O'Connor Daniel M, Qadri U, Arora VM (2017). Comparison of male vs female resident milestone evaluations by faculty during emergency medicine residency training. JAMA Intern Med.

[28] Sobol DL, Berfield KS, Shalhub S, Tatum RP, Yale LA, Perkins JD, Calhoun KE (2022). Exploring the influence of gender on surgical clerkship grades and test scores: A single institution, multisite comparison. J Surg Educ.

[29] Thackeray Erin W, Halvorsen Andrew J, Ficalora Robert D, Engstler Gregory J, McDonald Furman S, Oxentenko Amy S (2012). The effects of gender and age on evaluation of trainees and faculty in gastroenterology. Am J Gastroenterol.

[30] Fenoll-Brunet MR (2016). El concepto de internacionalización en enseñanza superior universitaria y sus marcos de referencia en educación médica. Educación Médica.

[31] Hidalgo DT, Pérez JAA, Rojas PAD, Aguilera NG (2022). The internationalization of higher education and medical universities as a source of development. EDUMECENTRO.

[32] Valdivieso MA, BURBANO VM, BURBANO AS (2020). Percepción de estudiantes universitarios colombianos sobre el efecto del confinamiento por el coronavirus, y su rendimiento académico. Espacios.

[33] García Prieto FJ (2022). Competencia digital del alumnado universitario y rendimiento académico en tiempos de COVID-19. Pixel-Bit.

[34] Kang L, Li Y, Hu S, Chen M, Yang C, Yang BX, Wang Y, Hu J, Lai J, Ma X, Chen J, Guan L, Wang G, Ma H, Liu Z (2020). The mental health of medical workers in Wuhan, China dealing with the 2019 novel coronavirus. Lancet Psychiatr.

[35] Lozano-Vargas A (2020). Impacto de la epidemia del Coronavirus (COVID-19) en la salud mental del personal de salud y en la población general de China. Rev Neuropsiquiatr.

[36] Shigemura J, Ursano RJ, Morganstein JC, Kurosawa M, Benedek DM (2020). Public responses to the novel 2019 coronavirus (2019-nCoV) in Japan: Mental health consequences and target populations. Psychiatry Clin Neurosci.

